# Awareness of Obesity-Related Cancers: A Complex Issue

**DOI:** 10.3390/ijerph19116617

**Published:** 2022-05-29

**Authors:** Zahra Mojtahedi, Shirin Farjadian

**Affiliations:** 1 Department of Health Care Administration and Policy, School of Public Health, University of Nevada, Las Vegas, NV 89154, USA; 2 Autophagy Research Center, Shiraz University of Medical Sciences, Shiraz 71348, Iran; 3 Department of Immunology, Shiraz University of Medical Sciences, Shiraz 71348, Iran; farjadsh@sums.ac.ir

## 1. Public Health, Cancer, and Obesity

Cancer rates are on the rise across the world, making the illness a public health crisis, particularly in developed countries where cancer has become a leading cause of death [1]. Despite a rise in the incidence of most cancers, lung cancer has been declining in recent years in the USA following public awareness of its association with tobacco smoking [2]. People have been choosing not to smoke to lower the risk of getting lung cancer. If it has worked for lung cancer, public awareness of other preventable cancer risk factors, notably obesity, should then successfully protect people from the related cancers. However, the obesity and cancer issue is complex, partly because of biological differences among people and also the possibility of an obesity stigma along with raising public awareness of obesity and cancer [3,4].

## 2. Obesity and Cancer

Obesity has been on the rise across the world, particularly in developed countries [5]. Being overweight or obese has been associated with a higher risk of getting 13 types of cancer [6,7]. These 13 types constitute 40% of all cancers diagnosed in the United States each year and are: adenocarcinoma of the esophagus, breast (in women who have gone through menopause), colon and rectum, uterus, gallbladder, upper stomach, kidneys, liver, ovaries, pancreas, thyroid, meningioma (a type of brain cancer), and multiple myeloma [6,7]. According to a World Health Organization (WHO) report, 30–50% of all cancer cases are preventable [8], and addressing preventable factors offers a long-term strategy for the control of cancer [9].

## 3. Public Awareness, Cancer, Obesity

Despite the association of obesity with cancer, public awareness of this association is low. A study in the UK has shown it as low as 25.4% among unprompted and 57.5% among prompted respondents [10]. One intervention for raising awareness of obesity and cancer can be through mass media. However, we should consider avoiding the obesity stigma, a situation that can cause mental health and other problems even in the workplace [3]. Actually, it might be more complicated to raise public awareness against obesity-related cancers than smoking-related cancers partly because of the reality of obesity stigma. The prevailing media messages that blame obese people for being lazy, weak-willed, and unsuccessful need to be replaced with messages that obesity is a chronic disease with a complex etiology [4], and advertisements should be further designed by a team of specialists from different areas.

## 4. Targeted Awareness, Cancer, and Obesity

Another intervention for fighting against obesity is raising the awareness of obesity and cancer among patients. A report from the UK in a sample of patients, weighted to be representative of the UK population aged 18+, indicated that only 17.4% of patients received advice about their weight, although 48.4% of the sample were overweight/obese [10]. Hypothetically, an effective way of fighting obesity-related cancers, while avoiding obesity stigma, can be a targeted approach to reach obese patients who are consulting health providers for other reasons. Today’s problem for a patient may not be related to obesity, but tomorrow’s problem might be. The effects on obese people by COVID-19 can be an example of their vulnerability to a range of illnesses [11], and health providers should open a recurring discussion with patients about obesity-related health cancers during routine consultations to prevent problems in the future.

## 5. Obesity and Cancer: A Complex Issue

In addition to raising awareness and educating people (individual level), public health issues should be addressed at other levels, such as community and policy levels and governmental initiatives [12]. For example, promoting exercise by providing a safe environment for jogging can be another intervention to reduce the obesity rate [13]. The complexity of fighting obesity might be partly the reason for the failure in fighting against the rising obesity rate. It should be noted that, in addition to an increased risk of certain cancers, obesity can drastically worsen the quality of life for cancer patients. Obese patients with cancer are frequently offered less supportive care, notably palliative care, which might be partly due to difficulty in helping overweight patients with their movements [14]. Therefore, public health professionals should come up with plans to address obesity to not only reduce cancer rates but also improve the quality of life.

## 6. Conclusions

Many known and unknown factors are associated with cancer. Education and awareness of preventable factors, notably obesity, as well as other interventions can be a vital step to lowering the risk of getting cancer. Health providers should target their communications with obese patients toward keeping a healthy weight even if patients’ current illnesses may not be related to obesity. Health providers should take advantage of public knowledge of COVID-19 and obesity to remind patients about the importance of a healthy weight. While considering the stigma of obesity, public awareness of obesity and cancer through mass media is another intervention. Community- and policy-level interventions should be taken into account as well. Fighting obesity-related cancers can be complex, but it should be urgently and vigorously addressed before the problem gets worse.
